# RNA-based therapeutics for neurological diseases

**DOI:** 10.1080/15476286.2021.2021650

**Published:** 2022-01-22

**Authors:** Karen Anthony

**Affiliations:** Centre for Physical Activity and Life Sciences, University of Northampton, Northampton, UK

**Keywords:** RNA, RNA therapeutics, exon skipping, antisense oligonucleotide, neurological disease, siRNA, RNA aptamer, RNA vaccine

## Abstract

RNA-based therapeutics have entered the mainstream with seemingly limitless possibilities to treat all categories of neurological disease. Here, common RNA-based drug modalities such as antisense oligonucleotides, small interfering RNAs, RNA aptamers, RNA-based vaccines and mRNA drugs are reviewed highlighting their current and potential applications. Rapid progress has been made across rare genetic diseases and neurodegenerative disorders, but safe and effective delivery to the brain remains a significant challenge for many applications. The advent of individualized RNA-based therapies for ultra-rare diseases is discussed against the backdrop of the emergence of this field into more common conditions such as Alzheimer’s disease and ischaemic stroke. There remains significant untapped potential in the use of RNA-based therapeutics for behavioural disorders and tumours of the central nervous system; coupled with the accelerated development expected over the next decade, the true potential of RNA-based therapeutics to transform the therapeutic landscape in neurology remains to be uncovered.

## Introduction

**Figure 1.**
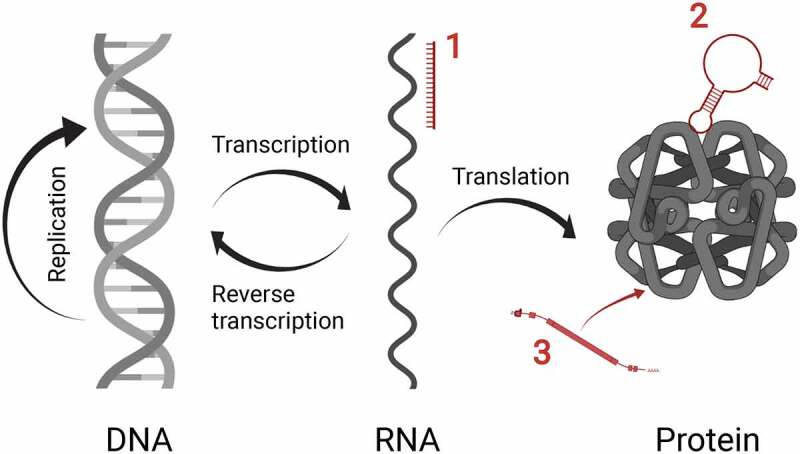
A schematic diagram illustrating the three broad categories of RNA-based therapeutics set against the central dogma of molecular biology. Group 1 targets RNA, group 2 uses RNA to target protein and group 3 uses mRNA to make protein. Illustration was created using BioRender.

The messenger RNA (mRNA)-based COVID-19 vaccines have thrust RNA-based therapeutics into the mainstream, but RNA drugs are far from new. Over 40 years of research is now culminating in the rapid expansion of these new classes of drugs, and approvals for diseases involving the nervous system are leading the way with ten approvals to date (Table 1). RNA-based therapeutics can be categorized into three groups according to their mechanism of action (Figure 1). The first group target nucleic acid using, for example, antisense oligonucleotides (AONs) or the RNA interference (RNAi) pathway. The second group of RNA drugs target proteins using RNA aptamers and the third group are mRNA drugs that encode proteins. Of these types, AONs are the most numerous; eight out of the ten RNA-based drug approvals for neurological diseases are of the AON modality (Table 1) and many more are under pre-clinical development. Although the most progress has been made for genetic diseases, the potential to develop RNA-based therapeutics to treat brain tumours, neurodegenerative diseases, stroke and behavioural disorders is strong, especially when you consider that such diseases are increasingly recognized as diseases of RNA metabolism [1]. Here, both the current and potential neurological applications for each type of RNA-based therapeutic are reviewed and their potential to change the therapeutic landscape across many diseases is highlighted.

**Table 1.** Approved RNA-based therapeutics for the treatment of neurological diseases

Drug	Market name	Target	Indication	First approval	Company
AON					
Fomivirsen	Vitravene	CMV gene UL123	Cytomegalovirus retinitis	FDA (1998)	Ionis Pharmaceuticals
Eteplirsen	Exondys 51	*DMD* exon 51	Duchenne muscular dystrophy	FDA (2016)	Sarepta Therapeutics
Nusinersen	Spinraza	*SMN2*	Spinal muscular atrophy	FDA (2016)	Biogen
Inotersen	Tegsedi	*TTR*	Hereditary transthyretin amyloidosis	FDA (2018)	Ionis Pharmaceuticals
Milasen	-	*MFSD8*	CLN7 Batten disease*	FDA (2018)	Boston Children’s Hospital
Golodirsen	Vyondys 53	*DMD* exon 53	Duchenne muscular dystrophy	FDA (2019)	Sarepta Therapeutics
Viltolarsen	Viltepso	*DMD* exon 53	Duchenne muscular dystrophy	FDA (2020)	NS Pharma
Casimersen	Amondys 45	*DMD* exon 45	Duchenne muscular dystrophy	FDA (2021)	Sarepta Therapeutics
siRNA					
Patisiran	Onpattro	*TTR*	Hereditary transthyretin amyloidosis	FDA (2018)	Alnylam Pharmaceuticals
RNA aptamer					
Pegaptanib	Macugen	VEGF-165	Age-related macular degeneration	FDA (2004)	OSI pharmaceuticals

*Customized drug designed to treat a single patient. Note that drugs approved for non-neurological conditions are excluded.

## Targeting nucleic acid

**Figure 2.**
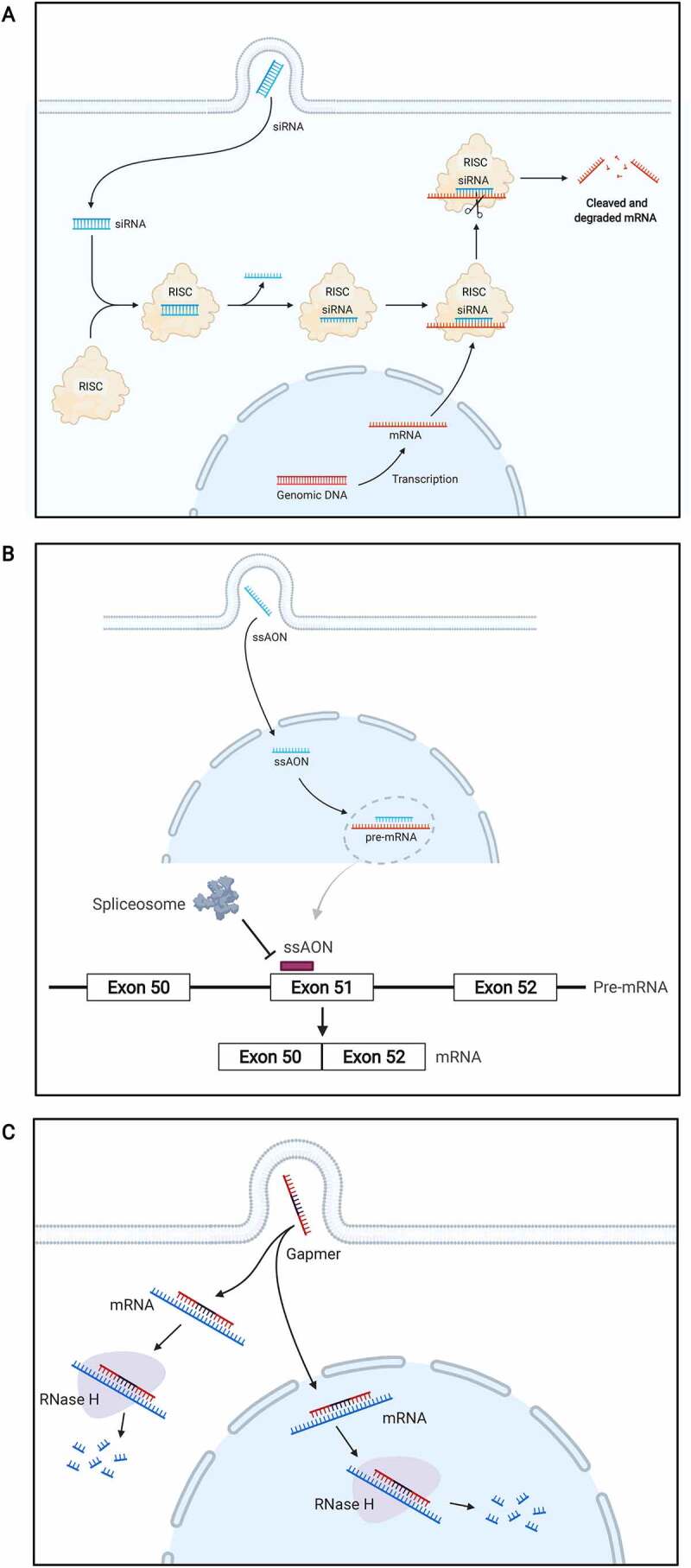
Targeting RNA using AONs and siRNA. (a) siRNA. After cellular uptake, a double-stranded siRNA is recruited to the RNA-induced silencing complex (RISC) and the passenger strand is removed. The guide strand then binds to its complementary mRNA before it is converted into protein, the RISC complex together with the siRNA cleaves the target mRNA thus silencing its protein production. (b) ssAONs. ssAONs are targeted to the nucleus where they bind to their target pre-mRNA. This binding sterically blocks the spliceosome and results in splicing modulation. In the example illustrated, the AON targets exon 51 of the dystrophin gene resulting in exon skipping. (c) Gapmer AONs. Gapmers can induce RNAse H-mediated cleavage of a target mRNA in both the nucleus and the cytoplasm. Illustration was adapted from ‘siRNA Nanoparticle Delivery System’, by BioRender.com (2021). Retrieved from https://app.biorender.com/biorender-templates.

The two major types of RNA-based therapeutics that target nucleic acid are the single-stranded AONs and the double-stranded small interfering RNA (siRNA) molecules that act through the RNAi pathway (Figure 2). Other more nuanced approaches that combine AONs with cellular machinery and traditional gene therapy delivery systems have also been developed such as spliceosome-mediated RNA *trans* splicing (SMaRT) [2] and uridine-rich 7 small nuclear RNA (U7 snRNA)-mediated gene therapy [3]. The goal for any of these approaches might be to modulate pre-mRNA splicing, alter target gene expression and/or edit RNA. Thus far, strategies have largely centred around correcting, or mitigating against the effect of, genetic mutations and progress has been largely driven by rare genetic disease research where an orphan drug designation affords a progressive view from regulatory agencies.

### AONs

**Figure 3.**
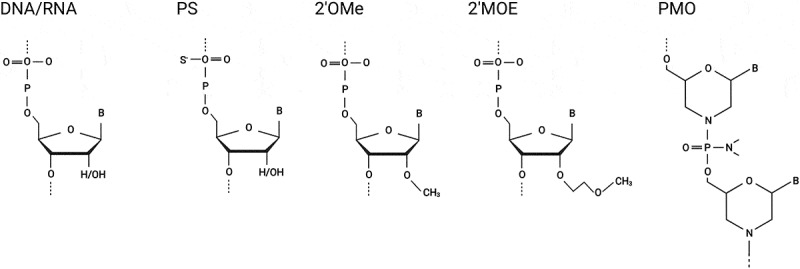
Common oligonucleotide chemistries. An unmodified DNA/RNA nucleotide is shown followed by the phosphorothioate (PS) backbone modification which replaces the original phosphodiester bond. A PS backbone is often used together with modifications to the 2ʹ-O position of the ribose; 2ʹ-O-methyl (2ʹOMe) and 2ʹ-O-methoxyethyl (2ʹMOE) modifications are illustrated. The uncharged phosphorodiamidate morpholino oligonucleotide (PMO) replaces the deoxyribose moiety of DNA with a 6-membered morpholino ring whilst retaining the normal nucleobases. Illustration was created using BioRender.

AONs are currently the largest modality of RNA-based therapeutics. Their rational design, chemistry and usage in cell, animal and clinical studies has been extensively reviewed elsewhere [4–6]. Briefly, AONs are short sequences of deoxynucleotides or deoxyribonucleotides which have been chemically modified to improve stability. The choice of chemistry largely depends on the desired application, all clinically approved AONs to date are either phosphorodiamidate morpholino oligomers (PMOs) or 2′-O-methoxyethyl (2ʹMOE) oligomers with a phosphorothioate (PS) backbone (2ʹMOE-PS) (Figure 3). PMOs are uncharged DNA analogues that bind to complementary RNA sequences through Watson–Crick base pairing and exert their effect by steric blockade [7]. 2ʹMOE is a common modification that adds a methyl group to the 2ʹhydroxyl of the ribose moiety and the PS backbone substitutes a sulphur atom for the non-bridging oxygen in the phosphate backbone. The PS backbone improves resistance to endonucleases and bioavailability but is known to also reduce affinity to the target RNA. Modifications at the 2ʹO position increase binding affinity and increase even further their nuclease resistance. Chimeric chemistries such as gapmers act by stimulating RNA cleavage via RNase H recruitment [8]. Gapmers contain a central region of DNA nucleotides flanked by for example, 2ʹO-modified sequences. Alternative and improved next-generation chemistries such as peptide-conjugated PMOs (pPMOs) have also emerged to improve efficiency and delivery to the target tissue [9]. The detailed mechanisms and challenges surrounding the delivery of oligonucleotide-based therapies have been recently reviewed [4]; delivery mechanisms to the brain are summarized here in Box 1. Regardless of their chemistry, therapeutic AONs are categorized according to their desired effect as outlined below and in Figure 2.

**Box 1.** Delivery to the brain

An efficient, and safe, delivery system is a current major hurdle. The blood-brain-barrier (BBB) prevents the passive diffusion of AONs without a delivery agent or brain-targeting conjugation. Direct intrathecal (IT) administration is therefore the most common delivery route to the central nervous system whereby AONs are administered into the subarachnoid space of the spinal cord [4]. IT administration can result in long-lasting AON concentrations since the BBB will prevent their peripheral circulation; as a result, a lower dose can be used. A subcutaneous port linked to an intrathecal catheter has been proposed as a safe alternative for repetitive lumbar punctures in individuals with spinal muscular atrophy but larger studies with long-term follow up are required [10]. Intraventricular injection can also be used to deliver AONs to the cerebrospinal fluid in the cerebral ventricles. Direct delivery to the eye using intravitreal injection or subretinal delivery is also well tolerated and intranasal delivery has also been achieved whereby the drug is transported into the brain along the rostral migratory stream [11,12]. Passage through the vascular BBB after systemic delivery can be achieved by exploiting existing receptor-mediated endocytosis pathways; for example, transferrin-targeting nanoparticles or antibodies complexed with AONs have been used in small animal model studies [13,14]. AONs conjugated to arginine-rich cell penetrating peptides (CPPs) are also known to cross the BBB in mice [15,16]. Naturally forming exosomes, and other nanoparticles, can be modified to display brain-targeting peptides and proteins such as the rabies virus glycoprotein (RVG) to enhance delivery across the BBB [17,18].

### Splice-switching AONs (ssAONs)

Genome-wide analysis of the tissue specificity of alternative splicing reveals that the brain makes the most complex use of alternative splicing and has the largest group of tissue-specific alternatively spliced isoforms [19]. Coupled with the fact that up to 50% of pathogenic mutations may affect splicing [20], the potential for therapeutic AONs to target splicing events in the brain is vast. ssAONs act via the steric block of intronic or exonic cis-regulatory elements to either force the inclusion or exclusion (skipping) of a target exon (Figure 2). ssAONs can target the splice sites themselves and/or exonic splicing enhancers (ESEs) or silencers (ESSs) or their intronic counterparts: intronic splicing enhancers (ISEs) and intronic splicing silencers (ISSs). The identification of these in and around the target region using bioinformatic tools is important for the rational design of efficient ssAONs and detailed guidelines for their use alongside other tools have been described [5]. ssAONs can also effectively block the use of cryptic splice sites or create novel donor or acceptor splice sites.

Most of the AONs approved for the treatment of neurological diseases to date are ssAONs (Table 1). Currently, these are Nusinersen for spinal muscular atrophy (SMA), the four ssAONs approved for the treatment of Duchenne muscular dystrophy (DMD) and Milasen (a customized ssAON for a single patient with Batten disease). The mechanism of action for Nusinersen is one of exon inclusion. SMA is a motor neurone disease caused by the loss or mutation of the *SMN1* gene which cannot be compensated for by its paralogue, *SMN2*, due to the almost total exclusion of exon 7. Nusinersen is a 2ʹMOE-PS ssAON that targets a strong ISS in intron 7 of *SMN2* (ISS-N1) to promote exon inclusion and the production of functional SMN protein from the *SMN2* gene [21,22]. Nusinersen is delivered to the central nervous system via intrathecal injection and is approved for the treatment of both paediatric and adult patients with all types of SMA [23–25]. After four initial loading doses, patients receive an indefinite maintenance dose three times a year.

In contrast, the parallel development of ssAONs for DMD utilized an exon skipping mechanism [6]. Duchenne is the most common type of muscular dystrophy and is caused by frame-shifting mutations in the *DMD* gene that prevent the full translation of its protein product, dystrophin [26]. Although dystrophin is essential for muscle function, it is lost throughout the whole body including the brain. A ‘DMD neuropsychiatric syndrome’ is common alongside the characteristic severe muscle wasting [27]. ssAONs for DMD are designed to skip exons that when removed would restore the reading frame and dystrophin protein production. Unlike for SMA, a single ssAON cannot treat all DMD patients due to the wide variability in patient deletions. Of the four FDA-approved drugs, Eteplirsen skips exon 51 and can treat approximately 13% of DMD patients in the Leiden DMD database [28]. Golodirsen and Viltolarsen both skip exon 53 and can treat approximately 8% of patients and Casimersen skips exon 45 to treat approximately 11% of patients in this database [29]. These DMD ssAONs are all PMOs and are delivered systemically via weekly intravenous infusions and all four compounds were approved based on evidence from surrogate outcome measures under the FDA’s accelerated approval pathway. The drugs are considered to be reasonably likely to provide a clinical benefit to patients, but confirmatory studies are required to verify and describe them. The development of exon skipping for DMD has therefore highlighted the importance of standardized and validated biochemical outcome measures to accurately quantify exon skipping and dystrophin expression [30–33]. Many other ssAONs to either skip different *DMD* exons using the same chemistry, or to provide enhanced delivery to target tissues such as the heart and brain using next-generation chemistries such as the pPMOs are currently progressing through pre-clinical and clinical development [9].

The approval of ssAONs for SMA and DMD has paved the way for other genetic diseases making the ssAONs a rapidly expanding drug modality. With advanced sequencing technologies the discovery of novel mutations amenable for such precision medicine is increasing. An example from neuromuscular field is the deep intronic splice defect in the collagen VI gene, *COL6A1*, that is causative of a collagen VI-related dystrophy, an extracellular matrix disorder [34]. This mutation inserts a pseudoexon which results in the production of a mutant collagen protein and defective collagen VI matrix assembly and function. A ssAON to skip the pseudoexon in patient-derived fibroblast cells effectively restored a wild-type collagen VI matrix assembly [34]. Such RNA sequencing initiatives used as a diagnostic tool have realized the development of n-of-1 AON-based therapies (Box 2.).

There is extensive pre-clinical and clinical research on the use of ssAONs to correct splicing defects in inherited retinal dystrophies [35–37]. For example, for Stargardt disease, ssAONs to correct intronic mutations which cause exon elongation or pseudoexons in the ATP-binding cassette transporter type 4 subfamily A, *ABCA4*, transcript show promise when tested in various cell models including induced pluripotent stem cell (iPSCs)-derived photoreceptor precursor cells [38,39]. The ssAON, sepofarsen, is under clinical development as a treatment for Leber congenital amaurosis [40] and an exon skipping ssAON is being investigated for the treatment of Usher syndrome type 2 [36,41].

ssAONs show promise also in the treatment of trinucleotide-repeat expansion disorders such as the spinocerebellar ataxias (SCAs). The SCAs comprise a heterogeneous group of approximately 45 autosomal dominant neurodegenerative diseases characterized by progressive ataxia, cognitive impairment, cerebellar atrophy and loss of cerebellar Purkinje cells and brainstem neurons [42,43]. The most common SCA subtypes are SCA1, 2, 3, 6 and 7 which are all nucleotide repeat expansion disorders. The long-term and/or complete downregulation of the resultant expanded proteins may not always be desirable given their important wild-type function(s). For SCA type 3, exon skipping can instead remove just the expanded exon (exon 10 of the *ATXN3* gene) to retain important functions such as ubiquitin binding and cleavage [44,45]. Repeated intracerebroventricular injections of such ssAONs in a SCA3 mouse model resulted in a beneficial effect on pathogenicity [45].

The rare developmental and epileptic encephalopathy, Dravet syndrome, is characterized by seizures, developmental delay and intellectual impairment. Most cases are caused by mutations in the *SCN1A* sodium channel gene. A ssAON to block the inclusion of a poison exon containing an in-frame stop codon is currently under clinical development after demonstrating efficacy in cell and animal models [46]. A single intracerebroventricular injection of the lead ssAON reduced seizures and sudden unexpected death in the mouse model.

The potential for ssAONs as therapeutics for the dementia-causing diseases is also gaining traction especially since defects in RNA metabolism are increasingly becoming apparent [1]. Targeting RNA at the source of pathogenesis may have a higher chance of success than targeting downstream pathways with different therapies. Given the role the proteolytic processing of the amyloid precursor protein (APP) plays in neurodegeneration, ssAONs have been investigated to modulate APP. ssAONs to skip APP exon 17 which encodes the γ-secretase cleavage site required for toxic amyloid-β production have shown efficacy in cell lines as well as in *in-vivo* [47,48]. ssAONs targeted against another major player in neurodegeneration, tau, are also under development. The tauopathies are characterized by the abnormal accumulation of the microtubule-associated protein tau, encoded by the *MAPT* gene. There are tau six isoforms that are split into two groups according to whether they have three microtubule-binding repeats (3 R) or four (4 R). 3 R and 4 R tau differ by the presence or absence of exon 10 which is extensively regulated [49–51]; many tauopathies are associated with an altered ratio of 3 R:4 R tau isoforms which in the normal adult human brain is one [49]. In frontotemporal dementia with parkinsonism linked to chromosome 17 (FTDP-17), several causative mutations destabilize a stem loop structure at the 3ʹ end of exon 10 resulting in increased exon 10 inclusion and 4 R tau [49]. Thus, altering the exon 10 splicing pattern using AONs may have a therapeutic benefit as has been shown using the SMaRT approach discussed below. Tau exon 10 inclusion can also be inhibited to reverse the effect of FTDP-17 mutations using bipartite AONs that flank the stem loop as well as by ssAONs targeting either splice site [52,53]. Further, exon skipping of tau exons 1, 5 or 7 alters the reading frame and will lead to a premature stop codon and a reduction in *MAPT* expression which may prevent aggregation. ssAONs that skip exon 5 efficiently reduced *MAPT* RNA and protein expression both *in-vitro* and in an *in-vivo* mouse model transgenic for the human *MAPT* gene [54].

**Box 2.** n-of-1 AON therapy

The potential for AONs to be used for individualized treatment has been realized. Milasen, a ssAON designed and developed to treat a single patient with a rare Batten disease-causing mutation went from concept to first injection in under ten months [55]. Batten disease is a broad group of severe neurodegenerative diseases characterized pathologically by defects in lysosomal function and clinically by visual loss, seizures and psychomotor impairment in early childhood [56]. The patient, Mila, whom the AON was named after, first experienced symptoms aged three and was treated aged seven. Unfortunately, she died of the disease aged 10 but fewer and milder seizures were reported after treatment by intrathecal injection [55]. A further n-of-1 ssAON, Atipeksen, has also been administered to a three-year-old patient with a specific ataxia-telangiectasia (AT) mutation. AT is caused by mutations in the ataxia telangiectasia mutated, *ATM*, gene and is associated with many multisystem defects in addition to the hallmark neurodegeneration [57]. Cryptic splice variants are common in AT [58] and Atipeksen creates a novel splice donor site so that a functional ATM protein can be made. Whilst no data on Atipeksen has been published, i*n-vitro* proof-of-concept for the use of ssAONs for additional AT mutations has been demonstrated [59]. Another example comes from a 25-year-old patient, Jaci, with amyotrophic lateral sclerosis (ALS) who advocated to receive an experimental drug for the disease that also killed her twin sister. ‘Jacifusen’, delivered intrathecally, was developed to treat her specific form of ALS which was caused by the P525L fused in sarcoma (FUS) gene mutation. After being reported that Jaci showed improved symptoms before she died, the drug is currently in phase III clinical trials with up to 63 other patients worldwide [60]. The rapidly emerging field of n-of-1 AON therapies and n-of-1 trials is however not well supported by current drug development processes and the regulatory conditions vary worldwide [61–63]. Robust guidance and standards are required to allow the effective scaling and measurement of the benefits of this approach across diverse disease types. To this end an AON treatment registry and the inclusion of generic outcome measures to allow an aggregated analysis of individual trials has recently been proposed [63].

### AONs that alter gene expression

AONs that do not modulate splicing alter target gene expression through other means. Most commonly, AONs in this category are designed to downregulate protein expression and reduce mutant protein toxicity. The first AON to be approved in 1998, Fomivirsen, specifically inhibits the replication of the human cytomegalovirus (CMV) by binding to the CMV gene UL123. Fomivirsen was used in the treatment of cytomegalovirus retinitis in immunocompromised patients but was taken off the market in the early 2000’s due to a drastic reduction in CMV cases after the development of highly active antiretroviral therapy [64].

A common modality for reducing protein expression is the gapmer AON which stimulates RNA cleavage via the recruitment of RNase H to a DNA-RNA duplex (Figure 2) [8]. There are many examples in both pre-clinical and clinical development. From the neuromuscular diseases field, gapmer AONs have shown efficacy for collagen VI-related congenital muscular dystrophy where they can selectively supress the expression of mutant allele transcripts and restore functional protein production [65]. Similarly, studies in myotonic dystrophy type 1 (DM1) have shown some efficacy for gapmer AONs to induce the degradation of mutant *DMPK* transcripts [66,67], however after only small insignificant changes were observed in a phase I/IIa clinical trial by Ionis Pharmaceuticals on ISIS-DMPKRx, this work was halted in favour of exploring further improved next-generation AON chemistries for DM1 treatment. The inhibition of aberrant *DUX4* expression in the adult muscular dystrophy, facioscapulohumeral muscular dystrophy (FSHD) has also been investigated as a potential therapeutic strategy. PMO AONs have shown efficacy in supressing *DUX4* expression in cell models as well as patient muscle xenografts in mice through targeting the *DUX4* polyadenylation signal [68,69]. More recently, locked nucleic acid (LNA) gapmers have been used to achieve knockdown of *DUX4* and show improvements in muscle fusion and structure *in-vitro* and efficient uptake and efficacy *in-vivo* further demonstrating the potential for AON therapy for FSHD [70]. An additional AON, IONIS-DNM2-2.5_RX_ targeting the dynamin 2 protein is also in development for the treatment of centronuclear myopathy, a rare congenital myopathy characterized by the abnormal localization of nuclei in the centre of skeletal muscle cells. The myopathy can be reversed in mice using AON-mediated knockdown of dynamin 2 [71].

The example of using ssAONs to remove a polyglutamine-expanded exon in SCA3 was discussed above. The trinucleotide repeat disorders are also good candidates for AON targeting designed to downregulate toxic protein expression and reduce aggregation. In the case of SCA1, SCA2, SCA3 and SCA7 this has been investigated in mouse models where AONs delivered via intracerebroventricular injection reduced disease protein levels and improved phenotypes highlighting significant promise for AON therapy in this group of diseases [72–77]. For Huntington’s disease (HD), also a trinucleotide repeat disorder, multiple AONs designed to reduce the production of the huntingtin protein have been developed with promising results from early clinical trials [78–80]. However, Tominersen developed by Roche in partnership with Ionis Pharmaceuticals and two AONs targeting single nucleotide polymorphisms (SNPs) in the mutant allele developed by Wave Life Sciences were disappointingly and unexpectedly halted at phases III and I/II respectively after it was concluded the potential benefits did not outweigh the risks [81]. Tominersen supresses wild-type as well as mutant huntingtin which may have played a role and the AONs from Wave likely did not have an efficient enough delivery to significantly lower levels of mutant huntingtin. The field awaits news from further analysis of the data from these trials.

In Parkinson’s disease (PD), a key pathological feature accompanying the loss of dopaminergic neurones in the substantia nigra is the cytoplasmic accumulation of α-synuclein, encoded by the *SNCA* gene. It is suggested that decreasing *SNCA* expression could delay disease onset or modify progression; this hypothesis has been tested using AONs. An amido-bridged nucleic acid (AmNA)-modified AON efficiently reduced *SNCA* mRNA and protein levels *in-vitro* and was efficiently delivered to the mouse brain via intracerebroventricular injection resulting in an amelioration of neurological defects in a PD mouse model expressing human wild-type *SNCA* [82]. Cole *et al*. with Ionis Pharmaceuticals have also tested such an approach and demonstrated efficacy in the non-human primate brain after intrathecal injection [83]; an AON, ION464, is currently being tested in clinical trials for patients with multiple system atrophy where the aberrant accumulation of α-synuclein is also prominent. Ionis Pharmaceutics have an additional AON in clinical development for PD (ION859) targeting Leucine Rich Repeat Kinase 2 (LRRK2). *LRRK2* is commonly mutated in PD and its increased activity is associated with pathogenesis; LRRK2 AONs have been shown to prevent the formation of α-synuclein inclusions in a PD mouse model [84]. An alternative, and novel, application of AON technology in PD comes from the suppression of the RNA-binding protein, PTBP1, to switch cell fate in-situ and repopulate lost neurones [85]. Here, the depletion of PTB through the AON targeting of *PTBP1* results in the conversion of astrocytes to functional dopaminergic neurones which reverses disease phenotype in a mouse model of PD [85].

ssAONs targeting tau were discussed above, but like for SCA3, AONs to reduce overall tau expression are also under investigation. This comes at a time where other high-profile tau-targeting treatments are returning disappointing results in clinical trials and there is uncertainty over how central tau is to neurodegeneration; supressing tau using AONs will help to shed light in this area. A phase 1b trial by Biogen and Ionis Pharmaceuticals testing IONIS-MAPT_RX_ for the treatment of Alzheimer’s disease has reported a robust time and dose-dependent reduction in total tau and phospho-tau in the cerebrospinal fluid; results from mouse and non-human primates were also encouraging but the effects on cognition are as yet unknown [86].

Amyotrophic lateral sclerosis (ALS), has not escaped the rise of investigative AON therapies. Tofersen is an AON developed by Ionis Pharmaceutics in partnership with Biogen and targets superoxide dismutase 1 (SOD1) to reduce its expression [87]. SOD1 mutations are a common and well-understood cause of familial ALS thought to result in a toxic gain-of-function. Delivered intrathecally to SOD1 familial ALS patients, an AON against SOD1 was well tolerated in early trials [87] but it was recently announced that Tofersen did not meet its primary efficacy endpoint in a phase III trial, though reduced disease progression was apparent in secondary and exploratory endpoints. Ionis have other AONs in their ALS pipeline including ION363 (otherwise known as Jacifusen, Box 2) and IONIS-C9_RX_. ION363 and IONIS-C9_RX_ target FUS and mutant chromosome 9 open reading frame 72 (C9ORF72) respectively. A hexanucleotide repeat expansion of C9ORF72 is the most common cause of familial ALS, producing toxic RNA foci and dipeptide proteins [88]. Wave Life Sciences also have an investigational stereopure AON targeting the C9ORF72 expansion, WVE-004, which is delivered via intrathecal injection and substantially reduced repeat-containing *C9orf72* transcripts and dipeptide repeat proteins whilst preserving normal protein expression in transgenic mice [89].

The polyneuropathy and protein misfolding disorder, hereditary transthyretin-mediated amyloidosis (hTTR) is caused by the abnormal breakdown of the transthyretin (TTR) protein which deposits as amyloid fibrils in various organs and tissues including frequently in the peripheral nervous system, the deposits ultimately lead to organ failure and death within five to 15 years of disease onset [90]. The AON Inotersen (marketed as Tegsedi and developed by Ionis Pharmaceuticals) has been approved for the treatment of hTTR in adults by weekly subcutaneous injection. Inotersen is a 2ʹMOE-PS gapmer that inhibits TTR production to reduce the build up of amyloid throughout the body. Another AON, Eplontersen is also in clinical development for all types of TTR amyloidosis. Eplontersen is a second-generation ligand-conjugated AON designed to reduce TTR production [91].

The leukodystrophy Alexander disease (AxD) is a rare condition affecting myelin sheath and is most often caused by gain-of-function mutations in glial fibrillary acidic protein (GFAP) which lead to the overproduction and toxic accumulation of GFAP in protein inclusions called Rosenthal fibres. ION373 is an AON under development by Ionis Pharmaceuticals which targets GFAP mRNA to inhibit its production. A striking reversal of Rosenthal fibres and long-lasting elimination of GFP throughout the brain and spinal cord was observed after intracerebroventricular injection in an AxD mouse model [92].

AONs to reduce mutant protein expression in the eye also show promise. The AON QR-1123 aims to restore vision in patients with RHO-associated autosomal dominant retinitis pigmentosa. QR-1123 is an allele-selective AON that targets the P23H mutation in the rhodopsin (RHO) gene to remove mutant transcripts and is currently in phase I/II clinical trials [37].

### SMaRT

**Figure 4.**
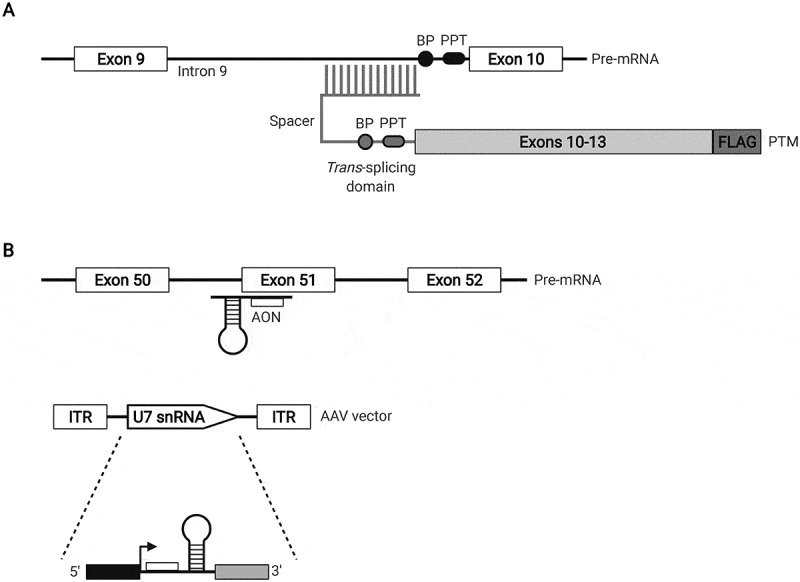
RNA-based gene therapy strategies. A) SMaRT is depicted in the context of the *MAPT* gene. A pre-*trans*-splicing molecule (PTM) is used to reprogram a mutant *MAPT* pre-mRNA transcript. The PTM contains the wild-type coding sequence of *MAPT* exons 10–13. A FLAG sequence at the 3ʹ end allows the detection of *trans*-spliced products. The PTM binding domain is complementary to the 3ʹ end of *MAPT* intron 9 and the PTM contains a branch point (BP), a polypyrimidine tract (PPT), an AG dinucleotide acceptor site and a spacer sequence separating the binding domain and branch point. B) U7snRNA-mediated exon skipping of the *DMD* gene using exon 51 as an example. An AON targeting exon 51 is indicated alongside the structure of the U7 snRNA cassette which is inserted between two inverted terminal repeats (ITRs) encoded by an AAV delivery vector. The U7 snRNA sequence is under the control of the natural U7 promoter (black box); the 3ʹ downstream elements are represented by the white box. Illustration was created using BioRender.

An elegant alternative to delivering short naked AONs to modify splicing or alter target gene expression is to use the specificity of an AON to repair an endogenous RNA species, expressed under endogenous transcriptional control, through a *trans*-splicing reaction. Spliceosome-mediated RNA *trans*-splicing, or SMaRT, is an RNA reprogramming technology that creates a hybrid mRNA through a *trans*-splicing reaction mediated by the spliceosome between the 5ʹ splice site of an endogenous target pre-mRNA and the 3ʹ splice site of an exogenously delivered pre-*trans*-splicing RNA molecule, or PTM (Figure 4) [2,93]. A typical PTM comprises a domain binding to an intron of the targeted RNA and a coding sequence corresponding to the new 3ʹ end of the reprogrammed mRNA and is delivered to cells by transfection of an expression vector, or by viral transduction. *Trans*-splicing is particularly suitable for the correction of dominant gain-of-function mutations; in this case, a corrected transcript is expressed while simultaneously, the mutant form is down-regulated. Possible *trans*-splicing on targets other than the intended target is possible but is unlikely to represent a major problem as many such products will be lost through nonsense-mediated decay [94]. In the context of neurological disorders, SMaRT has been used to successfully reduce the size of the CUG track in the *DMPK* transcript, which is expanded in myotonic dystrophy [95]. For SMA, SMaRT has been used to incorporate exon 7 in the transcript from the *SMN2* gene in SMA patient fibroblasts and in a mouse model of SMA, resulting in increased levels of full-length SMN protein [96–98]. In the latter case, reprogramming of *SMN2* mRNA resulted in an improved phenotype and longer survival [97]. Exon 10 usage in the neuronal *MAPT* gene transcript, encoding the microtubule-associated protein tau, can also be modulated using SMaRT [99]. SMaRT can correct splicing defects due to pathogenic *MAPT* mutations causing frontotemporal dementia with parkinsonism linked to chromosome 17 (FTDP-17) [100]. SMaRT can efficiently modulate *MAPT* alternative splicing in human-derived neurones [101] and can significantly reduce neurodegenerative pathology and improve cognitive impairment *in-vivo* in a mouse model of tauopathy [102]. Thus, there is substantial evidence to demonstrate that a neuronal transcript can be reprogrammed using SMaRT and improve pathology *in-vivo*.

### U7 snRNA-mediated gene therapy

Another more nuanced alterative to standard AONs takes advantage of the U-rich small nuclear ribonucleoproteins (snRNPs) which mediate pre-mRNA splicing. The U7 snRNP however does not play a role in splicing but rather processes the 3ʹend of replication-dependent histone mRNAs. The U7 snRNP histone binding domain can be custom-modified to target the splicing of a gene of interest and delivered using traditional gene therapy vectors such as adeno-associated viral (AAV) vectors (Figure 4) [3]. This has been achieved to induce both single and multiexon skipping (up to three exons in a single vector) for DMD, with proof-of-concept obtained in mouse models [103,104]. A phase I/IIa study is currently underway in humans to test the systemic (intravenous) delivery of scAAV9.U7.ACCA. This molecule targets a rare duplication of *DMD* exon 2 leading to either mRNA containing a single copy of exon 2 or no copies at all, the latter resulting in a functional but N-terminally truncated protein isoform [105]. Planned three-month post infusion data showed apparent expression of full-length dystrophin in two patients [106]. A potential benefit of this approach, and SMaRT, are that as gene therapy tools they can ensure a one-time lifelong treatment as opposed to the repeated administration of AONs.

### siRNA

In principle many neurological and neurodegenerative disorders could be treated or greatly diminished via gene silencing, however delivery roadblocks have slowed progress in comparison to siRNA delivery elsewhere such as the liver [4]. The one approval in the neurological field to date is Patisiran (Alnylam Pharmaceuticals) for the systemic polyneuropathy, hTTR [107]. Patisiran is intravenously delivered using a lipid nanoparticle delivery system. But the blood-brain barrier will limit the effectiveness of this for most brain diseases which require direct or efficient delivery to the brain itself. As with other RNA drug modalities, chemical modifications and conjugates are playing a role in furthering the promise of siRNA therapeutics through improving the delivery, stability and durability of the siRNA. There is strong commitment and investment from pharmaceutical companies who are rapidly progressing the therapeutic development of brain-targeting siRNAs for many neurological disorders. Pre-clinical programmes include targets for the neurodegenerative diseases: Alzheimer’s disease, Parkinson’s disease and Huntington’s disease [108]. Unlike for AONs which to date have focused largely on correcting specific genetic mutations, siRNA-based therapy also has a strong potential to deliver treatments for a full range of neurological disorders including stroke and brain tumours.

Until recent advances, when directly injected into the brain, siRNA affects only nearby cells for a short duration, leading to the undesirable requirement for frequent administration. However, potent and sustained huntingtin gene silencing throughout the central nervous system in small and non-human primate animal models has now been achieved using a divalent chemical scaffold (di-siRNA) delivered via a single injection into the cerebrospinal fluid [109]. Di-siRNAs consist of two phosphorothioate siRNAs connected through a linker, the number of PS modifications appears critical and unlike studies using traditional siRNA Alterman *et al*. observed minimal evidence for immune stimulation demonstrating its high tolerability as well as minimal off-target effects. Alternative conjugated-based delivery systems are being pioneered by Alnylam Pharmaceuticals who have used the sugar molecule, N-acetylgalactosamine (GalNAc) to effectively target the liver, the company have presented on the development of a similarly novel conjugate system to target the central nervous system with data in non-human primates on the silencing of β-catenin, a component of the Wnt signalling pathway and have an siRNA targeting APP in the pipeline [108].

An siRNA that efficiently crosses the BBB, Gal-NP@siRNA, has shown to effectively target *BACE1* in an Alzheimer’s disease mouse model showing improved cognitive capacity [110]. Gal-NP@siRNA is described as a glycosylated triple-interaction stabilized polymeric siRNA nanomedicine and blood-brain barrier penetration is achieved via stimulating the recycling of the glucose transpoter-1 (Glut-1) which is overexpressed on the luminal membrane after fasting [110]. Another BBB-crossing siRNA technology, the XB^3^ platform developed by Bioasis works via peptide conjugation (a 12 amino acid peptide called MTfp) and receptor-mediated transcytosis. Proof-of-concept has been obtained for the silencing of *NOX4* which plays a role in ischaemic stroke [111]. After induction of ischaemic stroke, animals treated with this novel siRNA suffered significantly reduced infarcts and improved recovery [111].

Many studies have explored the potential for siRNA to treat glioblastoma, the most aggressive form of glioma. These include for example the use of synthetic protein nanoparticles to target signal transducer and activator of transcription 3 (STAT3) [112] and siRNAs targeting β-catenin and the epidermal growth factor receptor (EGFR) genes [113].

siRNA also holds promise for the many genetic diseases discussed above for AON therapy. For example, there are numerous studies on the development of siRNA for the SCAs using various experimental systems, of which siRNA for SCA3/Machado-Joseph disease (MJD) has seen the most extensive effort towards allele-specific gene silencing [72]. Similarly, there is extensive siRNA-based research in the ophthalmology field with several ongoing clinical trials for disorders such as glaucoma, dry eye syndrome, age-related macular degeneration and diabetic macular oedema [37].

## Targeting proteins

**Figure 5.**
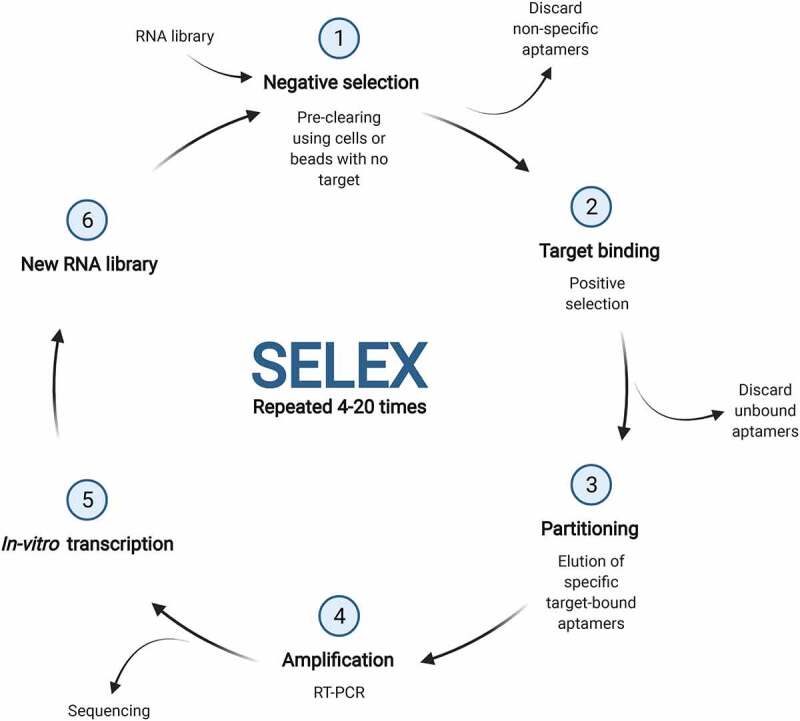
The SELEX process for the identification of RNA aptamers. The initial RNA library which is typically transcribed from DNA is bound to cells or beads with no target, or with structural analogues of the target, in a pre-clearing or negative selection step. After removing non-specific aptamers, the remaining pool is subjected to binding with the target and unbound aptamers are discarded. RT-PCR is used to amplify bound RNAs which can be identified via sequencing. A new library for the next round is generated using *in-vitro* transcription and the process repeated up to 40 times. Illustration was created using BioRender.

RNA aptamers are RNA molecules with a stable three-dimensional structure that are selected to bind a target with high affinity and specificity; targets include proteins, ions, whole cells and viruses [114]. Selection and isolation of RNA aptamers is performed from an RNA library using systematic evolution of ligands by exponential enrichment (SELEX) which allows for multiple successive rounds of evolution and screening (Figure 5) [115]. RNA aptamers have difficulty entering cells without specific cell-penetrating components and therefore primarily target cell surface molecules or those present in the blood stream [116]. The ability of aptamers to effectively bind cell-surface proteins however does makes them attractive delivery vehicles for siRNA. For example, aptamer-siRNA chimeras can be designed to inhibit a receptor function via the aptamer and silence a specific mRNA via the internalization of the siRNA [116,117]. Aptamers can also be fused together, such a bifunctional aptamer targeting the transferrin receptor and the epithelial cell adhesion molecule (EpCAM) has demonstrated proof-of-concept that such RNA aptamers can overcome the BBB to target brain disorders [118].

The specificity of RNA aptamers offers a functional advantage over small-molecule therapy owing to reduced toxicity and off-target effects and their immunogenicity is limited due to chemical modification [117]. Currently, there is one approved RNA aptamer targeting the nervous system; Pegaptanib (Macugen, approved by the FDA in 2004) for age-related macular degeneration. Pegaptanib is delivered by intravitreous injection and targets vascular endothelial growth factor (VEGF) to reduce pathological angiogenesis and vision loss [119]. After initial success however, pegaptanib has been somewhat superseded by the more effective monoclonal antibody therapies ranibizumab and bevacizumab. A major limitation of RNA aptamers is that their pharmacokinetic parameters such as renal excretion, hydrolysis and degradation are difficult to control which strongly influences efficacy [116]. Nonetheless, novel technologies and advances in aptamer design are driving forward the pre-clinical development of RNA-aptamers for the treatment of diverse neurological disorders such as multiple sclerosis (MS), stroke, epilepsy, prion disease and Alzheimer’s disease.

MS is characterized by pro-inflammatory leukocyte infiltration. Two therapeutic RNA aptamers targeting interleukin-17 (IL-17) and midkine (a heparin-binding growth factor) have both shown reduced inflammation in the experimental autoimmune encephalitis mouse model which resembles MS [120–122]. For ischaemic stroke, inhibition of the clotting factor IXa using an intravenously delivered RNA aptamer improved neurological function and reduced inflammation after cerebral ischaemia in mice [123]. To minimize the risk of life-threatening haemorrhage associated with such treatment, Blake *et al*. show that aptamer treatment in the context of intracranial haemorrhage can be reversed using a specific antidote, thus presenting a safer approach to the treatment of stroke. Similarly, an antidote-controlled RNA aptamer targeting Von Willebrand Factor has also been shown to significantly reduce stroke volumes in a dog model [124]. For epilepsy, RNA aptamers may also hold some significant advantages over current antiepileptic drugs which have high toxicity and suffer from poor targeting [125]. Targets for epilepsy RNA aptamers include the GABA receptor, the cell surface signalling receptor TrkB and the NMDA and AMPA receptors which all play recognized roles in epilepsy [125]. For neurodegeneration aptamers targeting β-secretase, Aβ fibrils and tau have been investigated for use primarily as diagnostic and imaging tools, but they may also hold promise as therapeutic agents [126–129]. For example, RNA aptamers targeting tau significantly inhibited tau oligomerisation and reduced neurotoxicity and dendritic spine loss in primary hippocampal neurons in tauopathy cell models [126]. Furthermore, RNA aptamers have been isolated that show specificity for insoluble prion protein (PrP) and have potential for the therapeutic prevention of PrP formation in humans [130,131].

## Making protein

**Figure 6.**
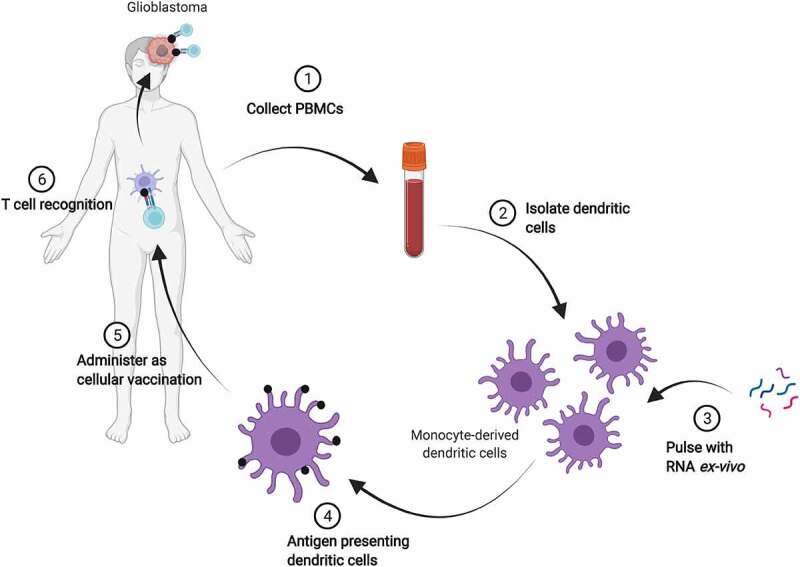
RNA-pulsed dendritic cell vaccine therapy for glioblastoma. After resection, total tumour RNA, or RNA encoding a specific tumour antigen, is ‘pulsed’ *ex-vivo* into dendritic cells derived from patient peripheral blood mononuclear cells (PBMCs). The resulting antigen presenting dendritic cells are administered as a vaccine to enable an antigen-specific immune response against the targeted tumour expressed epitope. Illustration was adapted from ‘Personalized Cell Therapies to Combat COVID-19’, by BioRender.com (2021). Retrieved from https://app.biorender.com/biorender-templates.

Administering mRNA as a therapeutic to treat neurological disorders has several advantages such as being able to produce proteins in their native form in mature non-dividing cells, without having to enter the nucleus. However, like all RNA-based therapeutics, delivery poses a challenge since mRNA is not particularly stable and is strongly immunogenic. There are currently no approved mRNA drugs to treat neurological diseases and clinical research is limited; but spurred by the success of COVID-19 vaccines, renewed efforts are underway for example to develop an mRNA vaccine for glioblastoma immunotherapy [132,133]. Such vaccines are designed to make a protein that can trigger an immune response. In an alternative approach, the feasibility and safety of the use of RNA-pulsed dendritic cell vaccines has already been demonstrated for recurrent glioblastoma (Figure 6) [134,135]. Here, RNA obtained from brain tumour stem cells is ‘pulsed’ into dendritic cells *ex-vivo* and redelivered. A first in human phase I/II study (NCT04573140) of an RNA-loaded nanoparticle vaccine is also underway for glioblastoma [135].

More in line with the other RNA-based therapeutics approaches discussed thus far, the feasibility of using mRNA to treat a sensory nerve disorder has been demonstrated by Baba *et al*. who, using a novel polyplex nanomicelle carrier, delivered brain-derived neurotrophic factor (BDNF) expressing mRNA via daily intranasal administration to a mouse model of olfactory dysfunction. They showed increased neurological recovery of olfactory function as well as the repair of olfactory epithelium to a nearly normal architecture [136]. An mRNA delivery system to produce BDNF has also more recently been shown to effectively prevent ischaemic neuronal death in a transient global ischaemia rat model after intraventricular injection [137].

## Small, circular and long non-coding RNAs

Circulating endogenous non-coding RNAs such as miRNAs, circular RNAs (circRNAs) and long non-coding RNAs are increasingly recognized for their potential use as accessible biomarkers for the diagnosis and prognosis of CNS diseases. Underlying disease mechanisms often begin pre-symptomatically and earlier diagnosis could be achieved (without direct access to brain tissue) through profiling the expression of miRNAs and other RNA species which are secreted into circulation. For example, many individual studies show that Alzheimer’s disease and/or mild cognitive impairment can be differentiated from cognitively normal controls through peripheral miRNA biomarkers [138], though few studies have yet assessed the same miRNAs under standardized conditions.

Improvements in sequencing technologies and bioinformatic pipelines have also revealed the potential for circRNAs as biomarkers in, for example, Alzheimer’s and Parkinson’s diseases where their association with pathological processes is well established [139]. However, research on circRNAs in CNS disorders remains in its infancy largely due to challenges surrounding the accurate detection of circRNAs. Nonetheless, their potential as both therapeutic agents and targets is an emerging and relevant area of investigation for neurological diseases, especially since they are highly expressed in the brain [140]. The circular structure renders circRNAs resistant to enzyme digestion and they are secreted from the brain in exosomes into bodily fluids making them attractive biomarkers as well as novel RNA therapeutics [141]. As an example, *circDLGAP4* (a known *mir-143* sponge) is downregulated in the brains of a rodent stroke model as well as in the plasma of human acute ischaemic stroke patients [142]. The overexpression of *circDLGAP4* delivered using a lentivirus to the lateral brain ventricle ameliorated stroke outcomes and improved neurological function in a mouse model of stroke [142].

There is now a long-standing recognition of the value of miRNAs as biomarkers, but as with circRNAs they are also viable drug targets and therapeutic agents themselves. The expression of target genes can be increased or decreased using miRNA inhibitors (antisense oligonucleotides called antagomiRs) and synthetic miRNA mimics respectively. miRNA mimics and inhibitors have been identified for application in CNS injuries such as stroke, traumatic brain injury and spinal cord injuries [143]. Similarly, miRNA-based therapies are also in development for neurodegenerative diseases such as Alzheimer’s disease; however, an important consideration is their multi-targeting mode of action which necessitates extensive pre-clinical assessment [144]. Combined with delivery challenges, the clinical targeting of miRNAs is therefore less advanced than other RNA-based therapeutics [145]. Nonetheless, a number of pharmaceutical companies are trialling miRNA inhibitors for the treatment of for example glioblastoma (Regulus Therapeutics: RGLS5579, an AON to inhibit miR-10b) [146] and ALS (miRagen Therapeutics: MRG-107, a miR-155 inhibitor) [147] and the next decade promises to define the feasibilities of miRNA-based drugs for clinical use in neurological diseases.

## Safety and toxicology

The rapid development of new chemistries and delivery systems for RNA-based therapeutics warrants careful screening for unwanted on- and off-target side effects. There is limited information on the toxicological properties of RNA aptamers but hybridization-dependent and hybridization-independent effects are well documented for AONs [4]. Hybridization-dependent effects include on-target toxicology issues induced by the prolonged activity of the RNA therapeutic during long-term treatment. The complete or partial off-target hybridization of RNA drugs is also a strong concern especially when a shorter sequence length is used. This is a particular issue for siRNA and gapmer drug modalities since they are designed to knockdown gene expression which could have disastrous effects if unintended genes are also downregulated [148,149]. Off-target effects are less of a concern for ssAONs since they require specific binding to regulatory splicing elements at the target site.

Hybridization-independent effects are more common than the above on- and off-target effects caused by Watson–Crick base pairing. AON toxicities induced by RNA-protein interactions are common and depend on the delivery system and chemistry used [4]. For example, PS-modified antisense oligonucleotides have a high protein-binding affinity which is known to inhibit blood coagulation, activate complement pathways and in some instances cause thrombocytopenia [4,150,151]. Various RNA-based drug modalities are also known to stimulate the immune system through for example binding to Toll-like receptors; of note however, the neutral PMO appears to not stimulate the immune system [4]. Infusion-related reactions manifesting as flu-like symptoms have also been reported for nanoparticle delivery systems such as that used for Patisiran where patients are required to be pre-medicated to supress such effects.

Regardless of the chemistry used, after intravenous administration a very high concentration of AON is found in the liver and kidneys (high exposure organs) where it accumulates as basophilic granules. This accumulation is generally considered non-adverse since the effect is reversed when treatment is terminated. However, some gapmers do induce a more severe and sequence-specific acute liver toxicity in mice after only a single treatment [4,152]. Thus, besides the delivery challenge, ensuring the safety and a favourable toxicology profile for the many new RNA-based therapeutics under development for CNS disorders is paramount.

## Conclusions

The possibilities to develop RNA-based drug modalities to treat neurological diseases appear limitless. Whilst progress is rapid across basic, translational and clinical research for most disease categories, there remains significant untapped potential in the use of RNA-based therapeutics for behavioural disorders and tumours of the central nervous system. Efficient, and safe, delivery remains an important hurdle but there is precedent to overcome such challenges from for example the discovery of GalNAc conjugation for liver targeting. With the advent of n-of-1 therapies, we are likely to witness changes in the regulatory processes for individualized medicine to best accommodate the requirement for the rapid treatment of rare progressive brain diseases, as well as the need to collectively measure the success of this approach across many different disease types. In parallel to the excitement surrounding n-of-1 therapies, the field is fast emerging out of the rare genetic disease research communities and witnessing the testing of RNA-based drugs in more common and chronic conditions. This is an essential development since AON drug approvals to date have been based on testing only small numbers of patients. Thus, their true potential to transform the neurological therapeutic landscape is yet to be uncovered.

## Data Availability

There is no data underpinning this publication.

## References

[1] Anthony K, Gallo J-M. Aberrant RNA processing events in neurological disorders. Brain Res. 2010;1338:67–77. 20226177 10.1016/j.brainres.2010.03.008

[2] Mansfield SG, Chao H, Walsh CE. RNA repair using spliceosome-mediated RNA trans-splicing. Trends Mol Med. 2004;10:263–268. 15177190 10.1016/j.molmed.2004.04.007

[3] Gadgil A, Raczyńska KD. U7 snRNA: a tool for gene therapy. J Gene Med. 2021;23: e3321 . 33590603 10.1002/jgm.3321 PMC8243935

[4] Hammond SM, Aartsma‐Rus A, Alves S, et al. Delivery of oligonucleotide-based therapeutics: challenges and opportunities. EMBO Mol Med. 2021;13:e13243 . 33821570 10.15252/emmm.202013243 PMC8033518

[5] Aartsma-Rus A. Overview on AON design. Methods Mol Biol. 2012;867:117–129. 22454058 10.1007/978-1-61779-767-5_8

[6] Arechavala-Gomeza V, Anthony K, Morgan J, et al. Antisense oligonucleotide-mediated exon skipping for Duchenne muscular dystrophy: progress and challenges. Curr Gene Ther. 2012;12:152–160. 22533380 10.2174/156652312800840621

[7] Summerton J, Weller D. Morpholino antisense oligomers: design, preparation, and properties. Antisense Nucleic Acid Drug Dev. 1997;7:187–195. 9212909 10.1089/oli.1.1997.7.187

[8] Wahlestedt C, Salmi P, Good L, et al. Potent and nontoxic antisense oligonucleotides containing locked nucleic acids. Proc Natl Acad Sci U S A. 2000;97:5633–5638. 10805816 10.1073/pnas.97.10.5633 PMC25880

[9] Gait MJ, Arzumanov AA, McClorey G, et al. Cell-penetrating peptide conjugates of steric blocking oligonucleotides as therapeutics for neuromuscular diseases from a historical perspective to current prospects of treatment. Nucleic Acid Ther. 2019;29:1–12. 30307373 10.1089/nat.2018.0747 PMC6386087

[10] Flotats-Bastardas M, Hahn A, Schwartz O, et al. Multicenter experience with nusinersen application via an intrathecal port and catheter system in spinal muscular atrophy. Neuropediatrics. 2020;51:401–406. 33091940 10.1055/s-0040-1715481

[11] Curtis MA, Kam M, Nannmark U, et al. Human neuroblasts migrate to the olfactory bulb via a lateral ventricular extension. Science. 2007;315:1243–1249. 17303719 10.1126/science.1136281

[12] Scranton RA, Fletcher L, Sprague S, et al. The rostral migratory stream plays a key role in intranasal delivery of drugs into the CNS. PLoS One. 2011;6:e18711 . 21533252 10.1371/journal.pone.0018711 PMC3076435

[13] Lee HJ, Boado RJ, Braasch DA, et al. Imaging gene expression in the brain in vivo in a transgenic mouse model of huntington’s disease with an antisense radiopharmaceutical and drug-targeting technology. J Nucl Med. 2002;43:948–956 . 12097468

[14] Kozlu S, Caban S, Yerlikaya F, et al. An aquaporin 4 antisense oligonucleotide loaded, brain targeted nanoparticulate system design. Pharmazie. 2014;69:340–345. 24855824

[15] Du L, Kayali R, Bertoni C, et al. Arginine-rich cell-penetrating peptide dramatically enhances AMO-mediated ATM aberrant splicing correction and enables delivery to brain and cerebellum. Hum Mol Genet. 2011;20:3151–3160. 21576124 10.1093/hmg/ddr217 PMC3140820

[16] Hammond SM, Hazell G, Shabanpoor F, et al. Systemic peptide-mediated oligonucleotide therapy improves long-term survival in spinal muscular atrophy. Proc Natl Acad Sci U S A. 2016;113:10962–10967. 27621445 10.1073/pnas.1605731113 PMC5047168

[17] Yang T, Fogarty B, LaForge B, et al. Delivery of small interfering RNA to inhibit vascular endothelial growth factor in zebrafish using natural brain endothelia cell-secreted exosome nanovesicles for the treatment of brain cancer. AAPS J. 2017;19:475–486. 27882487 10.1208/s12248-016-0015-y

[18] Alvarez-Erviti L, Seow Y, Yin H, et al. Delivery of siRNA to the mouse brain by systemic injection of targeted exosomes. Nat Biotechnol. 2011;29:341–345. 21423189 10.1038/nbt.1807

[19] Lee CJ, Irizarry K. Alternative splicing in the nervous system: an emerging source of diversity and regulation. Biol Psychiatry. 2003;54:771–776. 14550676 10.1016/s0006-3223(03)00375-5

[20] López-Bigas N, Audit B, Ouzounis C, et al. Are splicing mutations the most frequent cause of hereditary disease? FEBS Lett. 2005;579:1900–1903. 15792793 10.1016/j.febslet.2005.02.047

[21] Singh NK, Singh NN, Androphy EJ, et al. Splicing of a critical exon of human survival motor neuron is regulated by a unique silencer element located in the last intron. Mol Cell Biol. 2006;26:1333–1346. 16449646 10.1128/MCB.26.4.1333-1346.2006 PMC1367187

[22] Singh NN, Howell MD, Androphy EJ, et al. How the discovery of ISS-N1 led to the first medical therapy for spinal muscular atrophy. Gene Ther. 2017;24:520. 28485722 10.1038/gt.2017.34 PMC5623086

[23] Mercuri E, Darras BT, Chiriboga CA, et al. Nusinersen versus sham control in later-onset spinal muscular atrophy. N Engl J Med. 2018;378:625–635. 29443664 10.1056/NEJMoa1710504

[24] Aragon-Gawinska K, Seferian AM, Daron A, et al. Nusinersen in patients older than 7 months with spinal muscular atrophy type 1. Neurology. 2018;91:e1312–8. 30158155 10.1212/WNL.0000000000006281

[25] De Vivo DC, Bertini E, Swoboda KJ, et al. Nusinersen initiated in infants during the presymptomatic stage of spinal muscular atrophy: interim efficacy and safety results from the phase 2 NURTURE study. Neuromuscul Disord. 2019;29:842–856. 31704158 10.1016/j.nmd.2019.09.007 PMC7127286

[26] Muntoni F, Torelli S, Ferlini A. Dystrophin and mutations: one gene, several proteins, multiple phenotypes. Lancet Neurol. 2003;2:731–740. 14636778 10.1016/s1474-4422(03)00585-4

[27] Naidoo M, Anthony K. Dystrophin Dp71 and the neuropathophysiology of Duchenne muscular dystrophy. Mol Neurobiol. 2020;57:1748–1767. 31836945 10.1007/s12035-019-01845-w PMC7060961

[28] Aartsma-Rus A, Fokkema I, Verschuuren J, et al. Theoretic applicability of antisense-mediated exon skipping for Duchenne muscular dystrophy mutations. Hum Mutat. 2009;30:293–299. 19156838 10.1002/humu.20918

[29] Van Deutekom JCT, Van Ommen GJB. Advances in Duchenne muscular dystrophy gene therapy. Nat Rev Genet. 2003;4:774–783. 14526374 10.1038/nrg1180

[30] Hiller M, Falzarano MS, Garcia-Jimenez I, et al. A multicenter comparison of quantification methods for antisense oligonucleotide-induced DMD exon 51 skipping in Duchenne muscular dystrophy cell cultures. PLoS One. 2018;13:e0204485 . 30278058 10.1371/journal.pone.0204485 PMC6168132

[31] Anthony K, Arechavala-Gomeza V, Taylor LE, et al. Dystrophin quantification: biological and translational research implications. Neurology. 2014;83:2062–2069. 25355828 10.1212/WNL.0000000000001025 PMC4248450

[32] Anthony K, Cirak S, Torelli S, et al. Dystrophin quantification and clinical correlations in Becker muscular dystrophy: implications for clinical trials. Brain. 2011;134:3547–3559. 22102647 10.1093/brain/awr291 PMC3235564

[33] Anthony K, Feng L, Arechavala-Gomeza V, et al. Exon skipping quantification by quantitative reverse-transcription polymerase chain reaction in Duchenne muscular dystrophy patients treated with the antisense oligomer eteplirsen. Hum Gene Ther Methods. 2012;23:336–345. 23075107 10.1089/hgtb.2012.117

[34] Bolduc V, Reghan Foley A, Solomon-Degefa H, et al. A recurrent COL6A1 pseudoexon insertion causes muscular dystrophy and is effectively targeted by splice-correction therapies. JCI Insight. 2019;4:e124403 . 10.1172/jci.insight.124403 PMC6483063 30895940

[35] Gerard X, Garanto A, Rozet JM, et al. Antisense oligonucleotide therapy for inherited retinal dystrophies. Adv Exp Med Biol. 2016;854:517–524. 26427454 10.1007/978-3-319-17121-0_69

[36] Xue K, MacLaren RE. Antisense oligonucleotide therapeutics in clinical trials for the treatment of inherited retinal diseases. Expert Opin Investig Drugs. 2020;29:1163–1170. 10.1080/13543784.2020.1804853 32741234

[37] Gupta A, Kafetzis KN, Tagalakis AD, et al. RNA therapeutics in ophthalmology - translation to clinical trials. Exp Eye Res. 2021;205:108482 . 33548256 10.1016/j.exer.2021.108482

[38] Garanto A, Duijkers L, Tomkiewicz TZ, et al. Antisense oligonucleotide screening to optimize the rescue of the splicing defect caused by the recurrent deep-intronic ABCA4 variant c.4539+2001G>A in stargardt disease. Genes (Basel). 2019;10:452 . 10.3390/genes10060452 PMC6628380 31197102

[39] Tomkiewicz TZ, Suárez-Herrera N, Cremers FPM, et al. Antisense oligonucleotide-based rescue of aberrant splicing defects caused by 15 pathogenic variants in ABCA4. Int J Mol Sci. 2021;22:4621. 33924840 10.3390/ijms22094621 PMC8124656

[40] Cideciyan AV, Jacobson SG, Ho AC, et al. Durable vision improvement after a single treatment with antisense oligonucleotide sepofarsen: a case report. Nat Med. 2021 275. 2021;27:785–789. 33795869 10.1038/s41591-021-01297-7 PMC8127404

[41] Dulla K, Slijkerman R, van Diepen HC, et al. Antisense oligonucleotide-based treatment of retinitis pigmentosa caused by USH2A exon 13 mutations. Mol Ther. 2021;29:2441–2455. 33895329 10.1016/j.ymthe.2021.04.024 PMC8353187

[42] Paulson HL. The spinocerebellar ataxias. J Neuro-Ophthalmol. 2009;29:227–237. 10.1097/WNO0b013e3181b416de PMC2739122 19726947

[43] Buijsen RAM, Toonen LJA, Gardiner SL, et al. Genetics, mechanisms, and therapeutic progress in polyglutamine spinocerebellar ataxias. Neurotherapeutics. 2019;16:263–286. 30607747 10.1007/s13311-018-00696-y PMC6554265

[44] Evers MM, Tran HD, Zalachoras I, et al. Ataxin-3 protein modification as a treatment strategy for spinocerebellar ataxia type 3: removal of the CAG containing exon. Neurobiol Dis. 2013;58:49–56. 23659897 10.1016/j.nbd.2013.04.019

[45] Toonen LJA, Rigo F, van Attikum H, et al. Antisense oligonucleotide-mediated removal of the polyglutamine repeat in spinocerebellar ataxia type 3 mice. Mol Ther Nucleic Acids. 2017;8:232–242. 28918024 10.1016/j.omtn.2017.06.019 PMC5504086

[46] Han Z, Chen C, Christiansen A, et al. Antisense oligonucleotides increase Scn1a expression and reduce seizures and SUDEP incidence in a mouse model of Dravet syndrome. Sci Transl Med. 2020;12:eaaz6100 . 32848094 10.1126/scitranslmed.aaz6100

[47] Chang JL, Hinrich AJ, Roman B, et al. Targeting Amyloid-β precursor protein, APP, splicing with antisense oligonucleotides reduces toxic amyloid-β production. Mol Ther. 2018;26:1539–1551. 29628304 10.1016/j.ymthe.2018.02.029 PMC5986716

[48] Daoutsali E, Hailu TT, Buijsen RAM, et al. Antisense oligonucleotide-induced amyloid precursor protein splicing modulation as a therapeutic approach for Dutch-type cerebral amyloid angiopathy. Nucleic Acid Ther. 2021;31:351–363. 34061681 10.1089/nat.2021.0005 PMC8823675

[49] D’Souza I, Schellenberg GD. Regulation of tau isoform expression and dementia. Biochim Biophys Acta - Mol Basis Dis. 2005;1739:104–115. 10.1016/j.bbadis.2004.08.009 15615630

[50] Chapple JP, Anthony K, Martin TR, et al. Expression, localization and tau exon 10 splicing activity of the brain RNA-binding protein TNRC4. Hum Mol Genet. 2007;16:2760–2769. 17725984 10.1093/hmg/ddm233

[51] Malmqvist T, Anthony K, Gallo J-M. Tau mRNA is present in axonal RNA granules and is associated with elongation factor 1A. Brain Res. 2014;1584:22–27. 24389033 10.1016/j.brainres.2013.12.033

[52] Kalbfuss B, Mabon SA, Misteli T. Correction of alternative splicing of tau in frontotemporal dementia and parkinsonism linked to chromosome 17. J Biol Chem. 2001;276:42986–42993. 11560926 10.1074/jbc.M105113200

[53] Peacey E, Rodriguez L, Liu Y, et al. Targeting a pre-mRNA structure with bipartite antisense molecules modulates tau alternative splicing. Nucleic Acids Res. 2012;40:9836–9849. 22844088 10.1093/nar/gks710 PMC3479178

[54] Sud R, Geller ET, Schellenberg GD. Antisense-mediated exon skipping decreases tau protein expression: a potential therapy for tauopathies. Mol Ther Nucleic Acids. 2014;3:e180 . 25072694 10.1038/mtna.2014.30 PMC4121519

[55] Kim J, Hu C, Moufawad El Achkar C, et al. Patient-customized oligonucleotide therapy for a rare genetic disease. N Engl J Med. 2019;381:1644–1652. 31597037 10.1056/NEJMoa1813279 PMC6961983

[56] Dolisca SB, Mehta M, Pearce DA, et al. Batten disease: clinical aspects, molecular mechanisms, translational science, and future directions. J Child Neurol. 2013;28:1074. 23838031 10.1177/0883073813493665 PMC3986921

[57] McKinnon PJ. ATM and ataxia telangiectasia. EMBO Rep. 2004;5:772–776. 15289825 10.1038/sj.embor.7400210 PMC1299121

[58] Teraoka SN, Telatar M, Becker-Catania S, et al. Splicing defects in the ataxia-telangiectasia gene, ATM: underlying mutations and consequences. Am J Hum Genet. 1999;64:1617–1631. 10330348 10.1086/302418 PMC1377904

[59] Du L, Pollard JM, Gatti RA. Correction of prototypic ATM splicing mutations and aberrant ATM function with antisense morpholino oligonucleotides. Proc Natl Acad Sci. 2007;104:6007–6012. 17389389 10.1073/pnas.0608616104 PMC1832221

[60] Dolgin E. News feature: gene therapy successes point to better therapies. Proc Natl Acad Sci. 2019;116:23866–23870. 31772140 10.1073/pnas.1918306116 PMC6883820

[61] Aartsma-Rus A. ‘N of 1ʹ therapies need a better model. Nat Med. 2021 276. 2021;27:939. 34031602 10.1038/s41591-021-01380-z

[62] Selker HP, Cohen T, D’Agostino RB, et al. A useful and sustainable role for N-of-1 trials in the healthcare ecosystem. Clin Pharmacol Ther. 2021;1 doi: 10.1002/cpt.2425. PMC9022728 34551122

[63] Synofzik M, van Roon-mom WM, Marckmann G, et al. Preparing n-of-1 antisense oligonucleotide treatments for rare neurological diseases in Europe: genetic, regulatory, and ethical perspectives. Nucleic Acid Ther. 2021;Sep 29 doi: 10.1089/nat.2021.0039. PMC9058873 34591693

[64] Grillone LR, Lanz R. Fomivirsen. Drugs Today (Barc). 2001;37:245–255. 12768225 10.1358/dot.2001.37.4.620590

[65] Marrosu E, Ala P, Muntoni F, et al. Gapmer antisense oligonucleotides suppress the mutant allele of COL6A3 and restore functional protein in Ullrich muscular dystrophy. Mol Ther Nucleic Acids. 2017;8:416–427. 28918041 10.1016/j.omtn.2017.07.006 PMC5537204

[66] Klein A, Dastidar S, Furling D, et al. Therapeutic approaches for dominant muscle diseases: highlight on myotonic dystrophy. Curr Gene Ther. 2015;15:329–337. 26122101 10.2174/1566523215666150630120537

[67] Wheeler TM, Leger AJ, Pandey SK, et al. Targeting nuclear RNA for in vivo correction of myotonic dystrophy. Nature. 2012;488:111. 22859208 10.1038/nature11362 PMC4221572

[68] Marsollier AC, Ciszewski L, Mariot V, et al. Antisense targeting of 3ʹ end elements involved in DUX4 mRNA processing is an efficient therapeutic strategy for facioscapulohumeral dystrophy: a new gene-silencing approach. Hum Mol Genet. 2016;25:1468–1478. 26787513 10.1093/hmg/ddw015

[69] Chen JCJ, King OD, Zhang Y, et al. Morpholino-mediated knockdown of DUX4 toward facioscapulohumeral muscular dystrophy therapeutics. Mol Ther. 2016;24:1405–1411. 27378237 10.1038/mt.2016.111 PMC5023379

[70] Lim KRQ, Bittel A, Maruyama R, et al. DUX4 transcript knockdown with antisense 2ʹ-O-methoxyethyl gapmers for the treatment of facioscapulohumeral muscular dystrophy. Mol Ther. 2021;29:848–858. 33068777 10.1016/j.ymthe.2020.10.010 PMC7854280

[71] Tasfaout H, Buono S, Guo S, et al. Antisense oligonucleotide-mediated Dnm2 knockdown prevents and reverts myotubular myopathy in mice. Nat Commun. 2017 8 ;15661. 28589938 10.1038/ncomms15661 PMC5467247

[72] Afonso-Reis R, Afonso IT, Nóbrega C. Current status of gene therapy research in polyglutamine spinocerebellar ataxias. Int J Mol Sci. 2021;22:4249. 33921915 10.3390/ijms22084249 PMC8074016

[73] Moore LR, Rajpal G, Dillingham IT, et al. Evaluation of antisense oligonucleotides targeting ATXN3 in SCA3 mouse models. Mol Ther Nucleic Acids. 2017;7:200. 28624196 10.1016/j.omtn.2017.04.005 PMC5415970

[74] Friedrich J, Kordasiewicz HB, O’Callaghan B, et al. Antisense oligonucleotide-mediated ataxin-1 reduction prolongs survival in SCA1 mice and reveals disease-associated transcriptome profiles. JCI Insight. 2018;3:e123193 . 10.1172/jci.insight.123193 PMC6238731 30385727

[75] Scoles DR, Meera P, Schneider MD, et al. Antisense oligonucleotide therapy for spinocerebellar ataxia type 2. Nature. 2017;544:362–366. 28405024 10.1038/nature22044 PMC6625650

[76] Kourkouta E, Weij R, González-Barriga A, et al. Suppression of mutant protein expression in SCA3 and SCA1 mice using a CAG repeat-targeting antisense oligonucleotide. Mol Ther Nucleic Acids. 2019;17:601–614. 31394429 10.1016/j.omtn.2019.07.004 PMC6695277

[77] Niu C, Prakash TP, Kim A, et al. Antisense oligonucleotides targeting mutant Ataxin-7 restore visual function in a mouse model of spinocerebellar ataxia type 7. Sci Transl Med. 2018;10:eaap8677 . 30381411 10.1126/scitranslmed.aap8677 PMC6411060

[78] Tabrizi SJ, Leavitt BR, Landwehrmeyer GB, et al. Targeting huntingtin expression in patients with Huntington’s disease. N Engl J Med. 2019;380:2307–2316. 31059641 10.1056/NEJMoa1900907

[79] Kordasiewicz HB, Stanek LM, Wancewicz EV, et al. Sustained therapeutic reversal of Huntington’s disease by transient repression of huntingtin synthesis. Neuron. 2012;74:1031. 22726834 10.1016/j.neuron.2012.05.009 PMC3383626

[80] Skotte NH, Southwell AL, Østergaard ME, et al. Allele-specific suppression of mutant huntingtin using antisense oligonucleotides: providing a therapeutic option for all Huntington disease patients. PLoS One. 2014;9:e107434 . 25207939 10.1371/journal.pone.0107434 PMC4160241

[81] Kwon D. Failure of genetic therapies for Huntington’s devastates community. Nature. 2021;593:180. 33963316 10.1038/d41586-021-01177-7

[82] Uehara T, Choong CJ, Nakamori M, et al. Amido-bridged nucleic acid (AmNA)-modified antisense oligonucleotides targeting α-synuclein as a novel therapy for Parkinson’s disease. Sci Rep. 2019 91. 2019;9:1–13. 31110191 10.1038/s41598-019-43772-9 PMC6527855

[83] Cole TA, Zhao H, Collier TJ, et al. α-Synuclein antisense oligonucleotides as a disease-modifying therapy for Parkinson’s disease. JCI Insight. 2021;6:e135633 . 10.1172/jci.insight.135633 PMC8021121 33682798

[84] Zhao HT, John N, Delic V, et al. LRRK2 antisense oligonucleotides ameliorate α-synuclein inclusion formation in a Parkinson’s disease mouse model. Mol Ther Nucleic Acids. 2017;8:508–519. 28918051 10.1016/j.omtn.2017.08.002 PMC5573879

[85] Qian H, Kang X, Hu J, et al. Reversing a model of Parkinson’s disease with in situ converted nigral neurons. Nat. 2020 5827813. 2020;582:550–556. 10.1038/s41586-020-2388-4 PMC7521455 32581380

[86] DeVos SL, Miller RL, Schoch KM, et al. Tau reduction prevents neuronal loss and reverses pathological tau deposition and seeding in mice with tauopathy. Sci Transl Med. 2017;9:eaag0481 . 28123067 10.1126/scitranslmed.aag0481 PMC5792300

[87] Miller TM, Pestronk A, David W, et al. An antisense oligonucleotide against SOD1 delivered intrathecally for patients with SOD1 familial amyotrophic lateral sclerosis: a phase 1, randomised, first-in-man study. Lancet Neurol. 2013;12:435–442. 23541756 10.1016/S1474-4422(13)70061-9 PMC3712285

[88] Barker HV, Niblock M, Lee YB, et al. RNA misprocessing in C9orf72-linked neurodegeneration. Front Cell Neurosci. 2017;11:195 . 28744202 10.3389/fncel.2017.00195 PMC5504096

[89] Liu Y, Dodart JC, Tran H, et al. Variant-selective stereopure oligonucleotides protect against pathologies associated with C9orf72-repeat expansion in preclinical models. Nat Commun. 2021 121. 2021;12:1–15. 33558503 10.1038/s41467-021-21112-8 PMC7870851

[90] Tozza S, Severi D, Spina E, et al. The neuropathy in hereditary transthyretin amyloidosis: a narrative review. J Peripher Nerv Syst. 2021;26:155–159. 33960565 10.1111/jns.12451 PMC8360044

[91] Ackermann EJ, Guo S, Booten S, et al. Clinical development of an antisense therapy for the treatment of transthyretin-associated polyneuropathy. Amyloid. 2012;19(Suppl 1):43–44. 22494066 10.3109/13506129.2012.673140

[92] Hagemann TL, Powers B, Mazur C, et al. Antisense suppression of glial fibrillary acidic protein as a treatment for Alexander disease. Ann Neurol. 2018;83:27–39. 29226998 10.1002/ana.25118 PMC5876100

[93] Puttaraju M, Jamison SF, Mansfield SG, et al. Spliceosome-mediated RNA trans-splicing as a tool for gene therapy. Nat Biotechnol. 1999;17:246–252. 10096291 10.1038/6986

[94] Rusconi A. Gene repair by means of trans-splicing: a delicate balancing act between efficiency and specificity? Mol Ther. 2001;4:162–163. 11545605 10.1006/mthe.2001.0452

[95] Chen HY, Kathirvel P, Yee WC, et al. Correction of dystrophia myotonica type 1 pre-mRNA transcripts by artificial trans-splicing. Gene Ther. 2009;16:211–217. 18923454 10.1038/gt.2008.150

[96] Coady TH, Shababi M, Tullis GE, et al. Restoration of SMN function: delivery of a trans-splicing RNA Re-directs SMN2 pre-mRNA splicing. Mol Ther. 2007;15:1471–1478. 17551501 10.1038/sj.mt.6300222

[97] Coady TH, Lorson CL. Trans-splicing-mediated improvement in a severe mouse model of spinal muscular atrophy. J Neurosci. 2010;30:126–130. 20053895 10.1523/JNEUROSCI.4489-09.2010 PMC2836862

[98] Coady TH, Baughan TD, Shababi M, et al. Development of a single vector system that enhances Trans-splicing of SMN2 transcripts. PLoS One. 2008;3:e3468 . 18941511 10.1371/journal.pone.0003468 PMC2565107

[99] Rodriguez-Martin T, Garcia-Blanco MA, Mansfield SG, et al. Reprogramming of tau alternative splicing by spliceosome-mediated RNA trans-splicing: implications for tauopathies. Proc Natl Acad Sci. 2005;102:15659–15664. 16230627 10.1073/pnas.0503150102 PMC1266082

[100] Rodriguez-Martin T, Anthony K, Garcia-Blanco MA, et al. Correction of tau mis-splicing caused by FTDP-17 MAPT mutations by spliceosome-mediated RNA trans-splicing. Hum Mol Genet. 2009;18:3266–3273. 19498037 10.1093/hmg/ddp264 PMC2722988

[101] Lacovich V, Espindola SL, Alloatti M, et al. Tau isoforms imbalance impairs the axonal transport of the amyloid precursor protein in human neurons. J Neurosci. 2017;37:58–69. 28053030 10.1523/JNEUROSCI.2305-16.2016 PMC6705673

[102] Espíndola SL, Damianich A, Alvarez RJ, et al. Modulation of Tau Isoforms Imbalance Precludes Tau Pathology and Cognitive Decline in a Mouse Model of Tauopathy. Cell Rep. 2018;23:709–715. 29669277 10.1016/j.celrep.2018.03.079

[103] Goyenvalle A, Wright J, Babbs A, et al. Engineering multiple U7snRNA constructs to induce single and multiexon-skipping for Duchenne muscular dystrophy. Mol Ther. 2012;20:1212–1221. 22354379 10.1038/mt.2012.26 PMC3369406

[104] Goyenvalle A, Vulin A, Fougerousse F, et al. Rescue of dystrophic muscle through U7 snRNA-mediated exon skipping. Science. 2004;306:1796–1799. 15528407 10.1126/science.1104297

[105] Simmons TR, Vetter TA, Huang N, et al. Pre-clinical dose-escalation studies establish a therapeutic range for U7snRNA-mediated DMD exon 2 skipping. Mol Ther Methods Clin Dev. 2021;21:325–340. 33898631 10.1016/j.omtm.2021.03.014 PMC8047432

[106] Waldrop DM, Lawlor DM, Vetter TM, et al. LATE BREAKING NEWS ORAL PRESENTATION: LBO 3 Expression of apparent full-length dystrophin in skeletal muscle in a first-in-human gene therapy trial using the scAAV9.U7-ACCA vector. Neuromuscul Disord. 2020;30:S166–7.

[107] Suhr OB, Coelho T, Buades J, et al. Efficacy and safety of patisiran for familial amyloidotic polyneuropathy: a phase II multi-dose study. Orphanet J Rare Dis. 2015;10:109 . 26338094 10.1186/s13023-015-0326-6 PMC4559363

[108] Sheridan C. Billion-dollar deal propels RNAi to CNS frontier. Nat Biotechnol. 2019;37:702–704. 31267098 10.1038/d41587-019-00014-7

[109] Alterman JF, Godinho BMDC, Hassler MR, et al. A divalent siRNA chemical scaffold for potent and sustained modulation of gene expression throughout the central nervous system. Nat Biotechnol. 2019 378. 2019;37:884–894. 31375812 10.1038/s41587-019-0205-0 PMC6879195

[110] Zhou Y, Zhu F, Liu Y, et al. Blood-brain barrier-penetrating siRNA nanomedicine for Alzheimer’s disease therapy. Sci Adv. 2020;6:eabc7031. 33036977 10.1126/sciadv.abc7031 PMC7546706

[111] Eyford BA, Singh CSB, Abraham T, et al. A nanomule peptide carrier delivers siRNA across the intact blood-brain barrier to attenuate ischemic stroke. Front Mol Biosci. 2021;8:611367 . 33869275 10.3389/fmolb.2021.611367 PMC8044710

[112] Gregory JV, Kadiyala P, Doherty R, et al. Systemic brain tumor delivery of synthetic protein nanoparticles for glioblastoma therapy. Nat Commun. 2020 111. 2020;11:1–15. 33173024 10.1038/s41467-020-19225-7 PMC7655867

[113] Wang K, Park JO, Zhang M. Treatment of glioblastoma multiforme using combination of siRNA targeting EGFR and β-catenin. J Gene Med. 2013;15:42. 23319157 10.1002/jgm.2693 PMC4167580

[114] Hermann T, Patel DJ. Adaptive recognition by nucleic acid aptamers. Science. 2000;287:820–825. 10657289 10.1126/science.287.5454.820

[115] Ellington AD, Szostak JW. In vitro selection of RNA molecules that bind specific ligands. Nat. 1990 3466287. 1990;346:818–822. 10.1038/346818a0 1697402

[116] Byun J. Recent progress and opportunities for nucleic acid aptamers. Life. 2021;11:193. 33671039 10.3390/life11030193 PMC7997341

[117] Zhou J, Bobbin ML, Burnett JC, et al. Current progress of RNA aptamer-based therapeutics. Front Genet. 2012;3:234. 23130020 10.3389/fgene.2012.00234 PMC3486975

[118] Macdonald J, Henri J, Goodman L, et al. Development of a bifunctional aptamer targeting the transferrin receptor and epithelial cell adhesion molecule (EpCAM) for the treatment of brain cancer metastases. ACS Chem Neurosci. 2017;8:777–784. 28010059 10.1021/acschemneuro.6b00369

[119] Ng EWM, Shima DT, Calias P, et al. Pegaptanib, a targeted anti-VEGF aptamer for ocular vascular disease. Nat Rev Drug Discov. 2006 52. 2006;5:123–132. 16518379 10.1038/nrd1955

[120] Ishiguro A, Akiyama T, Adachi H, et al. Therapeutic potential of anti-interleukin-17A aptamer: suppression of interleukin-17A signaling and attenuation of autoimmunity in two mouse models. Arthritis Rheum. 2011;63:455–466. 20967861 10.1002/art.30108

[121] Wang J, Takeuchi H, Sonobe Y, et al. Inhibition of midkine alleviates experimental autoimmune encephalomyelitis through the expansion of regulatory T cell population. Proc Natl Acad Sci U S A. 2008;105:3915–3920. 18319343 10.1073/pnas.0709592105 PMC2268842

[122] Muramatsu T. Midkine: a promising molecule for drug development to treat diseases of the central nervous system. Curr Pharm Des. 2011;17:410–423. 21375488 10.2174/138161211795164167 PMC3267162

[123] Blake CM, Wang H, Laskowitz DT, et al. A reversible aptamer improves outcome and safety in murine models of stroke and hemorrhage. Oligonucleotides. 2011;21:11–19. 21142878 10.1089/oli.2010.0262 PMC3043993

[124] Anderson C, Huttinger A, Wheeler D, et al. Abstract WP99: reversible VWF inhibitor reduces stroke volumes compared to TPA in canine model of large vessel occlusion stroke. Stroke. 2020;51:AWP99 .

[125] Zamay TN, Zamay GS, Shnayder NA, et al. Nucleic acid aptamers for molecular therapy of epilepsy and blood-brain barrier damages. Mol Ther Nucleic Acids. 2020;19:157. 31837605 10.1016/j.omtn.2019.10.042 PMC6920299

[126] Kim JH, Kim E, Choi WH, et al. Inhibitory RNA aptamers of tau oligomerization and their neuroprotective roles against proteotoxic stress. Mol Pharm. 2016;13:2039–2048. 27120117 10.1021/acs.molpharmaceut.6b00165

[127] Murakami K, Obata Y, Sekikawa A, et al. An RNA aptamer with potent affinity for a toxic dimer of amyloid β42 has potential utility for histochemical studies of Alzheimer’s disease. J Biol Chem. 2020;295:4870–4880. 32127399 10.1074/jbc.RA119.010955 PMC7152766

[128] Rentmeister A, Bill A, Wahle T, et al. RNA aptamers selectively modulate protein recruitment to the cytoplasmic domain of beta-secretase BACE1 in vitro. RNA. 2006;12:1650–1660. 16888322 10.1261/rna.126306 PMC1557694

[129] Ylera F, Lurz R, Erdmann VA, et al. Selection of RNA aptamers to the Alzheimer’s disease amyloid peptide. Biochem Biophys Res Commun. 2002;290:1583–1588. 11820803 10.1006/bbrc.2002.6354

[130] Proske D, Gilch S, Wopfner F, et al. Prion-protein-specific aptamer reduces PrPSc formation. ChemBioChem. 2002;3:717–725. 12203970 10.1002/1439-7633(20020802)3:8<717::AID-CBIC717>3.0.CO;2-C

[131] Rhie A, Kirby L, Sayer N, et al. Characterization of 2ʹ-fluoro-RNA aptamers that bind preferentially to disease-associated conformations of prion protein and inhibit conversion. J Biol Chem. 2003;278:39697–39705. 12902353 10.1074/jbc.M305297200

[132] Zhong H, Liu S, Cao F, et al. Dissecting tumor antigens and immune subtypes of glioma to develop mRNA vaccine. Front Immunol. 2021;12:3259. 10.3389/fimmu.2021.709986 PMC8429949 34512630

[133] Tang X, Zhang S, Fu R, et al. Therapeutic prospects of mRNA-based gene therapy for glioblastoma. Front Oncol. 2019;9:1208. 31781503 10.3389/fonc.2019.01208 PMC6857656

[134] Dunn-Pirio AM, Vlahovic G. Immunotherapy approaches in the treatment of malignant brain tumors. Cancer. 2017;123:734–750. 27875627 10.1002/cncr.30371

[135] Melnick K, Dastmalchi F, Mitchell D, et al. Contemporary RNA therapeutics for glioblastoma. Neuromolecular Med. 2021;Jun 8:1–5 . 10.1007/s12017-021-08669-9 PMC8186014 34101090

[136] Baba M, Itaka K, Kondo K, et al. Treatment of neurological disorders by introducing mRNA in vivo using polyplex nanomicelles. J Control Release. 2015;201:41–48. 25599855 10.1016/j.jconrel.2015.01.017

[137] Fukushima Y, Uchida S, Imai H, et al. Treatment of ischemic neuronal death by introducing brain-derived neurotrophic factor mRNA using polyplex nanomicelle. Biomaterials. 2021;270:120681 . 33517206 10.1016/j.biomaterials.2021.120681

[138] Wu HZY, Ong KL, Seeher K, et al. Circulating microRNAs as biomarkers of Alzheimer’s disease: a systematic review. J Alzheimer’s Dis. 2015;49:755–766. 10.3233/JAD-150619 26484928

[139] Zhang M, Bian Z. The emerging role of circular RNAs in Alzheimer’s disease and Parkinson’s disease. Front Aging Neurosci. 2021;13:426. 10.3389/fnagi.2021.691512 PMC8311738 34322012

[140] Rybak-Wolf A, Stottmeister C, Glažar P, et al. Circular RNAs in the mammalian brain are highly abundant, conserved, and dynamically expressed. Mol Cell. 2014;58:870–885. 10.1016/j.molcel.2015.03.027 25921068

[141] Holdt LM, Kohlmaier A, Teupser D. Circular RNAs as therapeutic agents and targets. Front Physiol. 2018;9:1262. 30356745 10.3389/fphys.2018.01262 PMC6189416

[142] Bai Y, Zhang Y, Han B, et al. Circular RNA DLGAP4 ameliorates ischemic stroke outcomes by targeting miR-143 to regulate endothelial-mesenchymal transition associated with blood–brain barrier integrity. J Neurosci. 2018;38:32–50. 29114076 10.1523/JNEUROSCI.1348-17.2017 PMC6705810

[143] Sun P, Liu DZ, Jickling GC, et al. MicroRNA-based therapeutics in central nervous system injuries. J Cereb Blood Flow Metab. 2018;38:1125–1148. 29708005 10.1177/0271678X18773871 PMC6434449

[144] Walgrave H, Zhou L, De Strooper B, et al. The promise of microRNA-based therapies in Alzheimer’s disease: challenges and perspectives. Mol Neurodegener. 2021;16:1–16. 34742333 10.1186/s13024-021-00496-7 PMC8572071

[145] Bonneau E, Neveu B, Kostantin E, et al. How close are miRNAs from clinical practice? A perspective on the diagnostic and therapeutic market. Electron J Int Fed Clin Chem Lab Med. 2019;30:114–127. PMC6599191 31263388

[146] Wang D, Liu K, Cattatossi G, et al. LN229 viability assay Lead #1 category assays PRECLINICAL DEVELOPMENT OF miR-10b. ANTAGONIST FOR THE TREATMENT OF GLIOBLASTOMA. 2018 .

[147] Dasgupta I, Chatterjee A. Recent advances in miRNA delivery systems. Methods Protoc. 2021;4:1–18. 10.3390/mps4010010 PMC7839010 33498244

[148] Fedorov Y, Anderson EM, Birmingham A, et al. Off-target effects by siRNA can induce toxic phenotype. RNA. 2006;12:1188–1196. 16682561 10.1261/rna.28106 PMC1484448

[149] Burel SA, Hart CE, Cauntay P, et al. Hepatotoxicity of high affinity gapmer antisense oligonucleotides is mediated by RNase H1 dependent promiscuous reduction of very long pre-mRNA transcripts. Nucleic Acids Res. 2015;44:2093–2109. 26553810 10.1093/nar/gkv1210 PMC4797265

[150] Henry SP, Novotny W, Leeds J, et al. Inhibition of coagulation by a phosphorothioate oligonucleotide. Antisense Nucleic Acid Drug Dev. 1997;7:503–510. 9361909 10.1089/oli.1.1997.7.503

[151] Henry SP, Beattie G, Yeh G, et al. Complement activation is responsible for acute toxicities in rhesus monkeys treated with a phosphorothioate oligodeoxynucleotide. Int Immunopharmacol. 2002;2:1657–1666. 12469940 10.1016/s1567-5769(02)00142-x

[152] Burdick AD, Sciabola S, Mantena SR, et al. Sequence motifs associated with hepatotoxicity of locked nucleic acid - Modified antisense oligonucleotides. Nucleic Acids Res. 2014;42:4882–4891. 24550163 10.1093/nar/gku142 PMC4005641

